# Identification and characterization of core cellulolytic enzymes from *Talaromyces cellulolyticus* (formerly *Acremonium cellulolyticus*) critical for hydrolysis of lignocellulosic biomass

**DOI:** 10.1186/s13068-014-0151-5

**Published:** 2014-10-09

**Authors:** Hiroyuki Inoue, Stephen R Decker, Larry E Taylor, Shinichi Yano, Shigeki Sawayama

**Affiliations:** Biomass Refinery Research Center, National Institute of Advanced Industrial Science and Technology, 3-11-32 Kagamiyama, Higashi-Hiroshima, Hiroshima 739-0046 Japan; Biosciences Center, National Renewable Energy Laboratory, 1617 Cole Boulevard, Golden, CO 80401 USA; Division of Applied Biosciences, Graduate School of Agriculture, Kyoto University, Oiwake-cho, Kitashirakawa, Sakyo-ku, Kyoto, 606-8502 Japan

**Keywords:** *Talaromyces cellulolyticus*, Lignocellulose, Cellulases, Pretreated corn stover, Enzymatic hydrolysis

## Abstract

**Background:**

Enzymatic hydrolysis of pretreated lignocellulosic biomass is an essential process for the production of fermentable sugars for industrial use. A better understanding of fungal cellulase systems will provide clues for maximizing the hydrolysis of target biomass. *Talaromyces cellulolyticus* is a promising fungus for cellulase production and efficient biomass hydrolysis. Several cellulolytic enzymes purified from *T. cellulolyticus* were characterized in earlier studies, but the core enzymes critical for hydrolysis of lignocellulosic biomass remain unknown.

**Results:**

Six cellulolytic enzymes critical for the hydrolysis of crystalline cellulose were purified from *T. cellulolyticus* culture supernatant using an enzyme assay based on synergistic hydrolysis of Avicel. The purified enzymes were identified by their substrate specificities and analyses of trypsin-digested peptide fragments and were classified into the following glycosyl hydrolase (GH) families: GH3 (β-glucosidase, Bgl3A), GH5 (endoglucanase, Cel5A), GH6 (cellobiohydrolase II, Cel6A), GH7 (cellobiohydrolase I and endoglucanase, Cel7A and Cel7B, respectively), and GH10 (xylanase, Xyl10A). Hydrolysis of dilute acid-pretreated corn stover (PCS) with mixtures of the purified enzymes showed that Cel5A, Cel7B, and Xyl10A each had synergistic effects with a mixture of Cel6A and Cel7A. Cel5A seemed to be more effective in the synergistic hydrolysis of the PCS than Cel7B. The ratio of Cel5A, Cel6A, Cel7A, and Xyl10A was statistically optimized for the hydrolysis of PCS glucan in the presence of Bgl3A. The resultant mixture achieved higher PCS glucan hydrolysis at lower enzyme loading than a culture filtrate from *T. cellulolyticus* or a commercial enzyme preparation, demonstrating that the five enzymes play a role as core enzymes in the hydrolysis of PCS glucan.

**Conclusions:**

Core cellulolytic enzymes in the *T. cellulolyticus* cellulase system were identified to Cel5A, Cel6A, Cel7A, Xyl10A, and Bgl3A and characterized. The optimized mixture of these five enzymes was highly effective for the hydrolysis of PCS glucan, providing a foundation for future improvement of the *T. cellulolyticus* cellulase system.

## Background

Lignocellulosic biomass is a potential sustainable source of fermentable sugars for industrial use. Enzymatic hydrolysis of the cellulose and hemicellulose fractions of lignocellulosic biomass to obtain fermentable sugars is enhanced by pretreatments that disrupt the rigid biomass structure, such as dilute acid, alkali, hot compressed water, and milling treatments [1–3]. The composition and structure of pretreated biomass varies widely depending on the biomass feedstock and the pretreatment technology. This diversity of pretreated biomass complicates the development of an efficient enzymatic hydrolysis process.

Cellulase preparations derived from industrial fungal strains of the genera *Trichoderma*, *Talaromyces*, and *Chrysosporium* are widely used for the hydrolysis of pretreated biomass [4]. Fungal cellulase systems generally contain a variety of cellulolytic enzymes to hydrolyze the cellulose and hemicellulose main chains, including two types of cellobiohydrolases (CBHI and CBHII), endo-1,4-β-glucanase, endo-1,4-β-xylanase, and β-glucosidase, and some accessory enzymes to complete the degradation of hemicellulose [5–7]. These enzymes work in a synergistic manner, each creating newly accessible sites on the lignocellulosic substrate for the others to act on. However, the composition of enzymes in native fungal cellulase systems is not necessarily suitable for the hydrolysis of pretreated biomass. The supplementation of cellulase preparations with additional enzymes, and the use of statistical modeling to create optimized mixtures from purified core cellulolytic enzymes, are useful strategies for maximizing the hydrolysis of biomass [8–13]. Such investigations promote further understanding and improvement of fungal cellulase systems for industrial applications.

*Talaromyces cellulolyticus* (formerly *Acremonium cellulolyticus*) is one of several promising fungi that are comparable to *Trichoderma reesei* in regards to efficient biomass hydrolysis and cellulase production [4,14–16]. A relatively high level of β-glucosidase activity has been observed in *T. cellulolyticus* [17]. A mutant strain of *T. cellulolyticus*, CF-2612, produced 18 g/L extracellular protein in shake flasks [18]. The properties of several purified cellulolytic enzymes from *T. cellulolyticus* were reported in earlier studies [19–24], however, their synergistic activities for the hydrolysis of lignocellulosic biomass is unknown. In addition, there is little information on the primary sequences of the purified enzymes and thus the details of the cellulase system of *T. cellulolyticus* remain unclear. We recently developed a starch-inducible homologous expression system in *T. cellulolyticus* to express cellulolytic enzymes [25]. A recombinant CBHI (Cel7A) and xylanases from *T. cellulolyticus* have been expressed in this system, characterized, and found to be equivalent in activity to those expressed in the wild-type strain [25–27].

In this study, to identify the core enzymes in the *T. cellulolyticus* cellulase system, we purified and characterized six cellulolytic enzymes critical for the hydrolysis of lignocellulosic biomass from strain CF-2612: β-glucosidase (Bgl3A), two endoglucanases (Cel5A and Cel7B), CBHII (Cel6A), CBHI (Cel7A), and xylanase (Xyl10A). The purified core enzymes were evaluated for the synergistic hydrolysis of corn stover pretreated with dilute sulfuric acid.

## Results

### Purification and identification of cellulolytic enzymes from *Talaromyces cellulolyticus*

**Figure 1:**
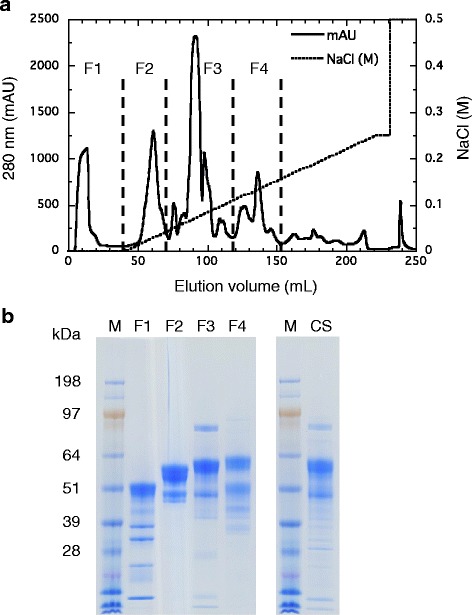
**Elution profile (a) and SDS-PAGE analysis (b) of** ***T. cellulolyticus*** **culture supernatant from a Resource Q column.** Protein peak fractions were eluted with a linear gradient of 0 to 0.25 M NaCl in 20 mM MES buffer (pH 6.5) and pooled into four groups (F1, F2, F3, and F4). Ten micrograms of protein were loaded per lane on SDS-PAGE analysis. CS, culture supernatant; M, protein marker; MES, 2-(N-morpholino) ethanesulfonic acid.

**Figure 2:**
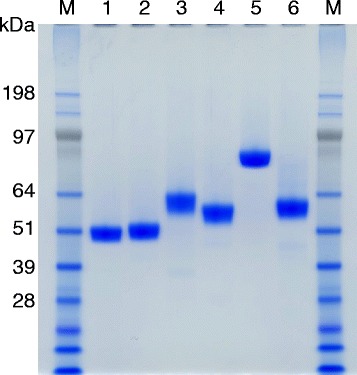
**SDS-PAGE analysis of purified enzymes.** Lanes: M, protein marker; 1, Xyl10A; 2, Cel5A; 3, Cel7B; 4, Cel6A; 5, Bgl3A; 6, Cel7A.

Cellulolytic enzymes involved in the hydrolysis of crystalline cellulose play a role as core enzymes in cellulase systems to degrade pretreated lignocellulosic biomass [8,12,13,28]. For purification of the core enzymes in the *T. cellulolyticus* cellulase system, we developed an assay for the synergistic hydrolysis of Avicel. First, synergistic activity for Avicel hydrolysis was determined for combinations of four groups (F1, F2, F3, and F4) of fractions obtained by Resource Q column chromatography of *T. cellulolyticus* culture supernatant (Figure 1). F2, containing Cel6A, showed synergism with F1, F3, and F4. F3, containing Cel7A and Bgl3A, was synergistic with F1 and F4, as well as F2. There was no synergism between F1 and F4. Thus, F2 and F3 were used to detect synergism for Avicel hydrolysis in fractions obtained by further chromatography of each group. Finally, six cellulolytic enzymes were purified to electrophoretically homogeneous forms (Figure 2). Tryptic peptide fragments obtained from the purified proteins were assigned to putative cellulolytic enzymes from *Talaromyces marneffei* or *Talaromyces stipitatus* in the National Center for Biotechnology Information (NCBI) database (Table 1) because putative genes from these fungi are known to have high similarity with those from *T. cellulolyticus* [29]. The genes corresponding to the *T. cellulolyticus* cellulolytic enzymes were found in our in-house *T. cellulolyticus* draft genome sequence. The glycosyl hydrolase (GH) families [30] of the six enzymes were determined from the deduced amino acid sequences and are as follows: GH10 for Xyl10A (from F1), GH6 for Cel6A (from F2), GH3 for Bgl3A (from F3), GH7 for Cel7A (from F3) and Cel7B (from F4), and GH5 for Cel5A (from F4).

**Table 1:** **Identification of peptides in purified enzymes**

**Enzyme**	**GH family**	**Molecular mass (theoretical, kDa)**	**Similar protein (NCBI GI number)**	**Species**	**Peptide sequences**	**Accession number**	**Reference**
Bgl3A	3	84.5	β-d-Glucoside glucohydrolase (GI:210068788)	*Talaromyces marneffei* ATCC 18224	K.GPCVGNTAAPSGISFPSLCIQDSPLGVR.Y	[FZ427523]	[31]
					R.YANPVTAFPAGTNAGMTWDR.T		
					K.GLGVHVQLGPVAGPLGK.I		
					K.HYIGNEQELNR.E		
					R.TLHELYLWPFADAVR.A		
					R.GCDTGTLAMGWGSGTCQFPYLTDPLTAIK.T		
					K.LSLAAGASGTATFDLTRR.D		
Cel5A	5	42.6	Endoglucanase, putative (GI:218723193)	*Talaromyces stipitatus* ATCC 10500	R.IPFAMER.M^a^	[HV540858]	[20]
					K.VIFDTNNEYHDMDETLVFNLNQAAIDGIR.G		
					R.VEGATAWLQANKK.L		
Cel6A	6	47.8	Cellobiohydrolase, putative (GI:242804399)	*Talaromyces stipitatus* ATCC 10500	K.AAEIPSFVWLDTAAK.V	[AB022429]	[23]
					K.VPTMGTYLANIEAANK.A		
					K.AGASPPIAGIFVVYDLPDR.D		
					R.DCAAAASNGEYTVANNGVANYK.A		
					K.AYPDVHTILIIEPDSLANMVTNLSTAK.C		
Cel7A	7	55.0	1,4-β-d-Glucan-cellobiohydrolyase, putative (GI:212538337)	*Talaromyces marneffei* ATCC 18224	K.SGGSCTTNSGAITLDANWR.W	[E39854]	[25]
					K.AGAQYGVGYCDSQCPR.D		
					R.YAGTCDPDGCDFNPYR.L		
					R.LGVTDFYGSGK.T		
					R.YYVQNGVVIPQPSSK.I		
Cel7B	7	50.9	Endoglucanase, putative (GI:210064489)	*Talaromyces marneffei* ATCC 18224	R.VYLLDPAGK.N^a^	[HV540856]	[20]
					K.TGTLTEIR.R		
Xyl10A	10	43.4	Endo-1,4-β-xylanase, putative (GI:242803213)	*Talaromyces stipitatus* ATCC 10500	K.GQCYAWDVVNEALNEDGTYR.Q	[AB796434]	[26]
					R.MTLPDTSALQTQQSTDYQTTTTACVQTK.G		

Theoretical molecular mass of the identified protein was based on the putative amino acid sequence including a signal sequence. Tryptic peptide fragments were identified by matching MS/MS spectra to *Aspergillus*, *Talaromyces*, and *Hypocrea* peptide sequences in the NCBI database. Cel5A and Cel7B peptides were identified by matching MALDI-TOF MS spectra with amino acid sequence translations of open reading frames in the *T. cellulolyticus* draft genome. ^a^Fragments were identified by both MS/MS spectra and MALDI-TOF MS spectra. GH, glycosyl hydrolase; MALDI-TOF MS, matrix-assisted laser desorption ionization time-of-flight mass spectrometry; MS/MS, tandem mass spectrometry.

The purification and homologous expression of Cel7A and Xyl10A have been reported previously [25,26]. Cel5A, Cel6A, and Cel7B were found to correspond to cellulase III-B (49 kDa), I (61 kDa), and III-A (58 kDa), respectively, according to their N-terminal sequences [20,23]. Bgl3A was recently purified and cloned [31]. Interestingly, all enzymes were found to have a carbohydrate-binding module 1; this is particularly rare in the case of β-glucosidase. This result appears to correlate with our initial purification method using the assay for synergistic hydrolysis of Avicel.

### Characterization of cellulolytic enzymes from *Talaromyces cellulolyticus*

The physicochemical and enzymatic properties of the purified enzymes are shown in Table 2. Cel7A had no activity on carboxymethyl cellulose (CMC) or p-nitrophenyl-β-d-glucoside, as reported previously [25]. Cellobiose-conjugated affinity chromatography was effective in removing the β-glucosidase contamination in Cel7A. Cel5A and Cel7B had significant activity not only on CMC but also on Avicel, in agreement with findings in earlier studies [20,23]. We found that Cel7B had higher specific activity on p-nitrophenyl-β-d-lactoside than Cel7A and trace activity on xylan, while Cel5A had no detectable activity on these substrates. Xylanase activity for endoglucanase has been reported for *T. reesei* Cel7B (endoglucanase I; EGI) [8,12].

**Table 2:** **Characteristics of purified enzymes**

**Enzyme**	**Molecular weight**		**Substrate specificity (U/mg)**						**Thermostability (** ***T***_**m**_ **, °C)**
	**(apparent, k)**								
	**SDS-PAGE**	**Gel filtration**	**Avicel**	**CMC**	**Birch xylan**	**PNP-lactose**	**PNP-glucose**	**PNP-xylose**	
Bgl3A	82.1	92.8	ND	ND	0.44	ND	10.70	0.10	66.0
Cel5A	50.4	61.6	0.26	17.70	ND	ND	0.05	ND	77.0
Cel6A	56.3	82.2	0.29	ND	0.87	ND	ND	ND	63.0
Cel7A	59.5	60.3	0.25	ND	ND	0.14	ND	ND	67.5
Cel7B	60.9	80.1	0.23	16.15	1.88	1.19	ND	ND	64.0
Xyl10A	49.8	44.1	ND	0.91	95.08	0.96	ND	0.31	82.0

*T*_m_ values were determined by fluorescence-based thermal shift assay as described in the Methods. CMC, carboxymethyl cellulose; ND, not detectable; PNP-glucose, p-nitrophenyl-β-d-glucoside; PNP-lactose, p-nitrophenyl-β-d-lactoside; PNP-xylose, p-nitrophenyl-β-d-xyloside.

Xyl10A had high specific activity on p-nitrophenyl-β-d-lactoside. This result is reasonable because some GH10 xylanases have activity on low molecular mass cellulose substrates, particularly aryl cellobiosides [32]. Minor endoglucanase activity (0.91 U/mg) was also observed for Xyl10A, whereas a recombinant Xyl10A purified previously had no detectable activity on CMC [26]. These results suggest that the Xyl10A purified in this study was contaminated with a trace amount of endoglucanase.

Comparison of the apparent molecular weights estimated by SDS-PAGE and gel filtration indicated that these proteins exist in a monomeric form (Figure 2, Table 2). The apparent molecular weights of the purified proteins were higher than the calculated molecular masses shown in Table 1, probably because of glycosylation. Carbohydrate contents in cellulases I (Cel6A), III-A (Cel7B), and III-B (Cel5A) have been estimated as 19.8%, 14%, and 16%, respectively [20,23]. This high glycosylation may occur because of O-glycosylation of serine and threonine residues in a flexible linker connecting a carbohydrate-binding module and a catalytic domain [33,34]. A thermal shift assay was used to determine the thermal midpoint (*T*_m_) values of the purified enzymes (Table 2). Cel5A and Xyl10A showed relatively high thermostability (77°C and 82°C, respectively). Cel6A had the lowest *T*_m_ value (63°C) among the enzymes; the relatively low thermostability of Cel6A may affect extended saccharification of lignocellulosic biomass at the higher end of conventional conditions (40 to 50°C) [35].

The synergism of the purified enzymes in the production of reducing sugars was examined by the Avicel hydrolysis assay. Both CBHs (Cel7A and Cel6A) displayed a pronounced synergism with all other enzymes (Table 3). Cel7A had the strongest synergism with Cel5A (237%), and Cel6A was most synergistic with Cel7B (174%). The synergism for the combination of Cel7A or Cel6A with Bgl3A was due to the hydrolysis of cellobiose to glucose, thereby relieving product inhibition. These results demonstrate the validity of using F2 (containing Cel6A) or F3 (containing Cel7A and Bgl3A) for the synergistic hydrolysis assay during purification of the cellulolytic enzymes. Unfortunately, however, the synergism between recombinant Xyl10A and Cel7A on Avicel was barely detectable in a subsequent study (Inoue, H., unpublished), suggesting that the results in the present study are due to trace contamination of Xyl10A with endoglucanase (Table 2).

**Table 3:** **Synergism of cellulolytic enzymes for the hydrolysis of Avicel**

**Enzyme**	**Synergism (%)**	
	**Cel6A**	**Cel7A**
Bgl3A	159	130
Cel5A	167	237
Cel6A	100	184
Cel7A	159	100
Cel7B	174	194
Xyl10A	142	168

Each enzyme sample was mixed with an equal amount of Cel6A or Cel7A (total 50 μg protein) and Avicel hydrolysis was measured after two hours at 45°C. Synergism was calculated based on the amount of reducing sugars in the reaction mixture relative to the amount produced by 50 μg of Cel6A or Cel7A.

### Synergism of cellulolytic enzymes of *Talaromyces cellulolyticus* for hydrolysis of pretreated corn stover

The purified enzymes were examined for synergistic hydrolysis of pretreated corn stover (PCS). Since the enzymes had been isolated according to their ability to hydrolyze crystalline cellulose, only the hydrolysis of PCS glucan was evaluated. Hydrolysis reactions were carried out at 45°C, pH 5, with 3% (w/v) PCS and a total protein loading of 2.55 mg/g glucan supplemented with Bgl3A. The ratio of the cellulase mixture to Bgl3A was fixed at 8:1 in this study. Bgl3A was used in excess to prevent inhibition by cellobiose and to yield glucose as the product of glucan hydrolysis [12]. All enzyme loadings were determined by bicinchoninic acid assay using bovine serum albumin as a standard.

**Figure 3:**
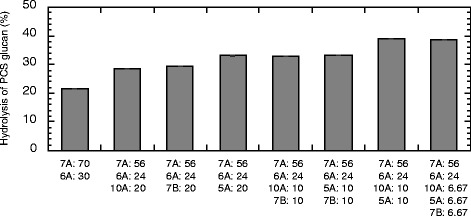
**Synergistic hydrolysis of PCS by the purified enzyme mixture.** Hydrolysis was carried out at pH 5, 45°C, with 3% (w/v) pretreated corn stover (PCS) and a protein loading of 2.55 mg/g glucan, consisting of 40 μg of the mixed cellulolytic enzymes and 5 μg of Bgl3A in a final volume of 1 mL. The proportions (%) of the mixed cellulolytic enzymes are noted under the columns. The percent hydrolysis of PCS glucan was calculated from the amount of glucose in the hydrolysate after 48 hours. 5A, Cel5A; 6A, Cel6A; 7A, Cel7A; 7B, Cel7B; 10A, Xyl10A.

The optimal PCS glucan hydrolysis by the mixture of Cel7A and Cel6A was observed at a ratio of approximately 7:3 (data not shown). Therefore, other enzymes were added to the 7:3 Cel7A:Cel6A mixture to evaluate synergistic effects. The addition of 20% Cel5A, Cel7B, or Xyl10A to the Cel7A:Cel6A mixture increased PCS glucan hydrolysis 1.55-, 1.37-, and 1.33-fold, respectively (Figure 3). The synergism between CBHs and endoglucanase (Cel5A or Cel7B) is consistent with the results shown in Table 3. The effect of Xyl10A might be due to exposure of the cellulose microfibrils by removal of the xylan in PCS, rather than contamination of Xyl10A with endoglucanase. Furthermore, the addition of 10% Cel5A or Cel7B to the Cel7A:Cel6A:Xyl10A mixture increased glucan hydrolysis 1.37- and 1.17-fold, respectively (Figure 3). The results suggest that Xyl10A and endoglucanase play different roles in the synergistic hydrolysis of PCS. Cel5A was found to be more synergistic with other enzymes than Cel7B. The addition of Cel7B to the Cel7A:Cel6A:Cel5A or Cel7A:Cel6A:Xyl10A:Cel5A mixture hardly affected glucan hydrolysis, suggesting that Cel5A and Cel7B have overlapping functions in the hydrolysis of PCS.

The combination of Cel7A, Cel6A, Xyl10A, and Cel5A was the most effective for hydrolysis of PCS glucan, producing a 1.82-fold increase in hydrolysis compared with the Cel7A:Ce6A mixture. These four enzymes and Bgl3A were defined as core cellulolytic enzymes of *T. cellulolyticus* and used to evaluate PCS hydrolysis in this study.

### Optimization of cellulolytic enzyme composition for pretreated corn stover hydrolysis

**Figure 4:**
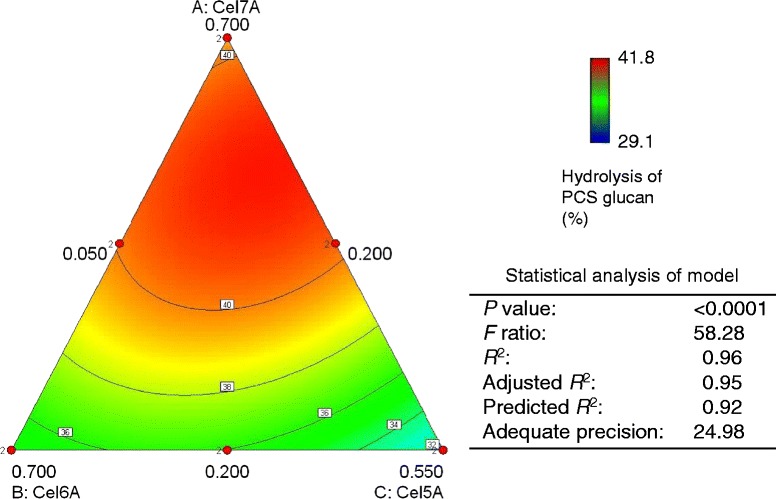
**Ternary plot and statistical analysis of the predictive model for hydrolysis of PCS glucan.** Analysis of the predictive model for PCS glucan hydrolysis was based on the data in Table 4. In the plot, Xyl10A has been fixed at a proportion of 0.05. The mixture of cellulolytic enzymes giving the highest predicted yield was Cel7A:Cel6A:Cel5A:Xyl10A (53:25:17:5). PCS, pretreated corn stover.

We determined statistically designed combinations of four core enzymes (Cel5A, Cel6A, Cel7A, and Xyl10A) for the hydrolysis of PCS glucan. Using an augmented quadratic model, the four-component experiment had 15 separate assay mixtures, each supplemented with Bgl3A. Based on the results described in the previous section, minimum proportions of 20% (Cel7A and Cel6A) and 5% (Cel5A and Xyl10A) were used as lower limits in designed mixtures. The reproducibility of the hydrolysis experiments at 48 hours was satisfactory, as standard deviations were generally less than 2% (n = 4) (Table 4). The best experimental mixture tested (combination 12) showed 41.6% glucan hydrolysis. The ternary diagram and statistical analyses of the models are given in Figure 4. A Cel7A:Cel6A:Cel5A:Xyl10A (53:25:17:5) mixture had the highest predicted yield, corresponding to 41.2% glucan hydrolysis. Analysis of variance (ANOVA) showed that the quadratic mixture model was significant (*F* =58.28, *P* <0.0001). The predicted *R*^2^ of 0.92 was in reasonable agreement with the adjusted *R*^2^ of 0.95. Adequate precision, a measure of the signal-to-noise ratio, was 24.98, indicating an adequate signal [8]. When the proportion of Xyl10A was increased to 10%, the optimal proportions of the other three enzymes were predicted to decrease in a similar way. These results suggest that Xyl10A has a different function from the other three enzymes in the synergistic hydrolysis of PCS.

**Table 4:** **Enzymatic hydrolysis of pretreated corn stover (PCS) by various mixtures of core cellulolytic enzymes**

**Combination number**	**Proportion of enzyme mixture**				**PCS glucan hydrolysis (%)**	
	**Cel7A**	**Cel6A**	**Cel5A**	**Xyl10A**	**Average**	**SD**
1	0.325	0.325	0.175	0.175	38.6	0.4
2	0.700	0.200	0.050	0.050	39.5	0.0
3	0.200	0.700	0.050	0.050	34.9	0.2
4	0.200	0.200	0.550	0.050	31.8	0.0
5	0.200	0.200	0.050	0.550	29.3	0.2
6	0.450	0.450	0.050	0.050	39.3	0.3
7	0.450	0.200	0.300	0.050	39.6	0.3
8	0.450	0.200	0.050	0.300	36.5	0.2
9	0.200	0.450	0.300	0.050	35.4	0.2
10	0.200	0.450	0.050	0.300	33.5	0.4
11	0.200	0.200	0.300	0.300	32.3	0.6
12	0.512	0.263	0.113	0.113	41.6	0.2
13	0.263	0.512	0.113	0.113	38.0	0.4
14	0.263	0.263	0.362	0.113	36.6	0.1
15	0.263	0.263	0.113	0.362	34.3	0.5

Hydrolysis was carried out at pH 5, 45°C, with 3% (w/v) PCS and a protein loading of 2.55 mg/g glucan, consisting of 40 μg of core enzyme mixture (Cel7A, Cel6A, Cel5A, and Xyl10A) and 5 μg of Bgl3A in a final volume of 1 mL. Percent hydrolysis of PCS glucan was calculated from the amount of glucose in the hydrolysate after 48 hours. SD, standard deviation.

### Evaluation of pretreated corn stover hydrolysis by the core cellulolytic enzyme mixture

**Figure 5:**
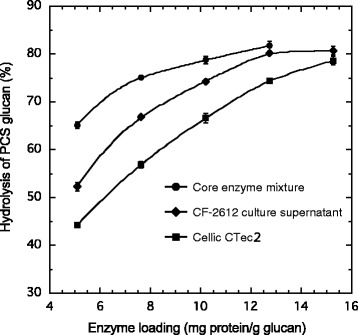
**Effect of enzyme loading on the hydrolysis of PCS glucan.** Hydrolysis was carried out at pH 5, 45°C, with 3% (w/v) PCS. The percent hydrolysis of PCS glucan was calculated from the amount of glucose in the hydrolysate after 48 hours. PCS, pretreated corn stover.

To compare the hydrolytic activity of the core enzyme mixture and crude enzymes, three different enzyme mixtures were evaluated at different total protein loadings for PCS glucan hydrolysis (Figure 5). The following enzyme preparations were compared: the core enzyme mixture Cel7A:Cel6A:Cel5A:Xyl10A:Bgl3A (46:25:13.5:4.5:11) approximating to the optimized composition (Figure 4), the crude enzyme from *T. cellulolyticus* CF-2612, and a commercial enzyme preparation (Novozymes NA., Franklinton, North Carolina, United States) from a genetically modified strain of *T. reesei*, Cellic CTec2, which has an increased level of β-glucosidase. The core mixture hydrolyzed PCS glucan more efficiently than the crude and commercial enzymes (Figure 5). The enzyme loading to achieve 75% hydrolysis was 7.5, 10.5, and 13 mg/g glucan for the core mixture, the crude enzymes, and the commercial preparation, respectively. This suggests that the core mixture was more potent for the degradation of cellulose in PCS than the crude and commercial enzymes, which contain extra enzymatic components. The lower degree of hydrolysis with the commercial enzyme preparation is probably due to the relatively low reaction temperature (45°C) because the optimum temperature for Cellic CTec2 is 50°C. To achieve 80% hydrolysis, which is necessary for an economical industrial process, further loading of 3.7 mg/g glucan was required for the core mixture, while an additional 2 mg/g glucan was required for the crude enzyme (Figure 5). This difference indicates that trace amounts of other enzymes such as hemicellulases are required for efficient hydrolysis of PCS glucan by the core enzymes.

## Discussion

Core enzymes in fungal cellulase systems are key factors in the economical saccharification of lignocellulosic biomass. It is well known that efficient hydrolysis of crystalline cellulose, a major component of lignocellulosic biomass, requires the synergistic activity of various types of cellulolytic enzymes. In this study, the core enzymes of the *T. cellulolyticus* cellulase system were purified and identified. Except for Bgl3A, these enzymatic properties have been reported previously [20,23,25,26]. We classified the purified enzymes into GH families based on primary structure and demonstrated that Cel7A, Cel6A, Cel5A, Xyl10A, and Bgl3A play important roles in the synergistic hydrolysis of PCS glucan.

The synergistic Avicel hydrolysis assay used in this study enabled the simultaneous purification of various cellulolytic enzymes (CBHs, endoglucanases, a xylanase, and a β-glucosidase) critical for the saccharification of crystalline cellulose. Some of these enzymes may be difficult to purify using a conventional Avicelase assay. On the other hand, the synergistic hydrolysis assay with F2 or F3 did not necessarily require high Avicelase activity of the target enzyme, and the synergistic activity was easily distinguishable from the activity of F2 or F3 alone. Furthermore, this assay discriminated between Cel7A and Cel6A during the purification process. Some minor isoforms of Cel7A and Cel6A, which have lower molecular weights than the major enzymes, were successfully purified using the synergistic hydrolysis assay with F2 and F3, respectively (Inoue, H., unpublished). The presence of Cel7A and Cel6A isoforms and/or degraded forms of the same protein has also been observed in two-dimensional electrophoretic analysis of the *T. reesei* secretome [36,37]. These isoforms may be readily purified using our synergistic hydrolysis assay with purified Cel6A or Cel7A.

The six purified cellulolytic enzymes were nearly consistent with the kinds of core enzymes proposed for the *T. reesei* cellulase system. Banerjee *et al.* reported the hydrolysis of ammonia fiber expansion (AFEX) treated corn stover using six core enzymes: Cel6A, Cel7A, Cel7B (EGI), β-glucosidase (GH3), β-xylosidase (GH3), and endo-β-1,4-xylanase (GH10) [8]. These proteins are abundant in the secretome of *T. reesei* RUT-C30 grown on AFEX-treated corn stover and in Spezyme CP, a commercial cellulolytic enzyme preparation [38]. *T. cellulolyticus* core enzymes have also been reported to be highly expressed in cellulose culture [26,39]. These results indicate that the major enzymes for cellulose hydrolysis are similar between *T. cellulolyticus* and *T. reesei*. This is one reasonable explanation for previous reports that the cellulase from *T. cellulolyticus* was effective for the hydrolysis of various pretreated lignocellulosic materials [14,16].

Xyl10A was found to play a significant role in the synergistic hydrolysis of PCS glucan regardless of the contamination by endoglucanase activity. Increases in cellulose digestibility of pretreated biomass have been associated with decreased xylan content [40]. Jeoh *et al.* reported that the removal of more than 80% of xylan, corresponding to 8% of xylan content in PCS treated with dilute acid, did not significantly improve digestion by purified Cel7A [41]. The PCS sample used in this study contained only 3.4% xylan; however, enzymatic xylan removal without increased pretreatment severity may produce newly accessible sites to improve digestion of PCS by CBHs. The proportion of Xyl10A in the optimized mixture for PCS hydrolysis corresponded to the lower limit (5%) in the experimental design (Figure 4). The relatively low proportion of Xyl10A is probably related to the low xylan content of the PCS. In glucan hydrolysis of AFEX-treated corn stover containing 22.4% xylan, the optimum proportion of xylanase (GH10) in a mixture of six *T. reesei* core enzymes, was estimated as 22% [8]. Furthermore, synergism between GH10 and GH11 xylanases for glucan hydrolysis of AFEX-treated corn stover has been reported for *T. reesei* core enzymes [42]. The genome of *T. cellulolyticus* possesses seven open reading frames with similarity to GH11 xylanase genes, and six of the corresponding enzymes have been characterized [27]. The roles of Xyl10A and the GH11 xylanases of *T. cellulolyticus* in the hydrolysis of lignocellulosic biomass should be evaluated in the future.

Cel5A, but not Cel7B, was selected as an endoglucanase in our core mixture for the hydrolysis of PCS. The two enzymes seemed to have overlapping endoglucanase activities, but hydrolysis of PCS was greater in mixtures with Cel5A than in mixtures with Cel7B (Figure 3). However, some contrasting results have been reported for the hydrolysis of lignocellulosic biomass using a *T. reesei* core mixture. Combinations of *T. reesei* Cel7A, Cel6A, Cel7B (EGI), and Cel5A (endoglucanase II; EGII) were used to degrade barley straw substrates subjected to three different pretreatments (water-soaked steam explosion, acid-soaked steam explosion, and hot water extraction), and the results showed that EGII activity is not required for efficient lignocellulose hydrolysis [13]. Billard *et al.* reported that EGI activity in the enzyme mixture was not compensated for by EGII activity for the hydrolysis of steam-pretreated wheat straw [11]. On the other hand, overlapping activity between EGI and EGII has been observed in the hydrolysis of AFEX-treated corn stover [9]. At a high solids content, EGI served as the key enzyme to rapidly reduce the viscosity of hydrothermally-pretreated wheat straw [43]. These reports indicate that the efficacy of Cel5A and Cel7B in the core enzyme mixture for the saccharification of lignocellulosic biomass depends on the pretreatment and the hydrolysis conditions.

The hydrolytic efficacy of the five-enzyme mixture (Cel7A:Cel6A:Cel5A:Xyl10A:Bgl3A) for PCS glucan surpassed that of the crude and commercial enzymes (Figure 5). The results suggest that the components of the *T. cellulolyticus* cellulase system have high potential for PCS saccharification. Furthermore, the optimized composition of core enzymes provides a starting point for improving the *T. cellulolyticus* cellulase system using molecular techniques. The main components of the core mixture are cellulases involved in the hydrolysis of crystalline cellulose; Xyl10A was the only hemicellulase included in the study. Supplementation with hemicellulases and rational design of cellulase and hemicellulase mixtures can help reduce cellulase loading and give higher overall hydrolysis yields [7,9,10,44]. Use of the core mixture will be helpful for finding the *T. cellulolyticus* hemicellulases critical for the overall hydrolysis of PCS.

## Conclusions

Six cellulolytic enzymes critical for hydrolysis of crystalline cellulose were purified from *T. cellulolyticus*, characterized, and classified into the following GH families: GH3 (β-glucosidase, Bgl3A), GH5 (endoglucanase, Cel5A), GH6 (CBHII, Cel6A), GH7 (CBHI and endoglucanase; Cel7A and Cel7B, respectively), and GH10 (xylanase, Xyl10A). These enzymes showed synergism for the hydrolysis of PCS glucan. The statistically designed combination of Cel7A, Cel6A, Xyl10A, Cel5A, and Bgl3A that was defined as the core cellulolytic enzyme mixture was highly effective for the hydrolysis of PCS glucan compared with a culture supernatant of *T. cellulolyticus* and a commercial cellulase preparation. The enzyme loading to achieve 80% hydrolysis was 11.2 mg/g glucan for the core mixture. These results suggest that the major components of the *T. cellulolyticus* cellulase system have high potential for PCS saccharification. The optimized composition of core enzymes will provide a foundation for future improvement of the *T. cellulolyticus* cellulase system using molecular techniques.

## Methods

### Pretreated corn stover sample

PCS was prepared with dilute sulfuric acid and steam in a pilot-scale continuous reactor [45]. The acidic pretreatment slurry (National Renewable Energy Laboratory PCS lot P080828CS) contained 17.1% (w/w) insoluble solids and was stored at 4°C prior to use. The average composition (% dry weight) of the insoluble solids was 58.9% glucan, 3.4% xylan, 0.7% arabinan, 0.5% galactan, 3.6% ash, and 20.8% lignin [45].

### Production of cellulolytic enzymes

*T. cellulolyticus* CF-2612 (FERM BP-10848) was grown in medium containing 5% (w/v) Solka-Floc (Fiber Sales & Development, Urbana, Ohio, United States) as described previously [18]. The whole broth was centrifuged at 13,500 × g and the resulting supernatant was filtered through a 0.22-μm polyether sulfone membrane (Thermo Scientific, Rockford, Illinois, United States) under sterile conditions. The culture filtrate containing cellulolytic enzymes was stored at 4°C.

### Purification of cellulolytic enzymes

Enzyme purification was carried out using an ÄKTAflpc chromatography system (GE Healthcare, Buckinghamshire, United Kingdom) at room temperature. The culture filtrate was desalted using a HiPrep 26/10 desalting column (GE Healthcare) equilibrated with 20 mM 2-(N-morpholino)ethanesulfonic acid buffer at pH 6.5 (buffer A). The desalted sample was applied to a Source 15Q anion-exchange column (GE Healthcare) equilibrated with the same buffer, and protein peaks were eluted with a linear gradient of 0 to 0.25 M NaCl. The protein peaks were divided into four groups (F1, F2, F3, and F4) in the chromatogram (Figure 1a), and the peak fractions in each group were pooled and concentrated by ultrafiltration over a membrane with a 10 kDa cutoff (Figure 1b).

The F1 sample, which included Xyl10A, was applied to a Source 15S cation-exchange column (GE Healthcare) equilibrated with 20 mM sodium acetate buffer at pH 4.0 (buffer B). The flow-through fractions were pooled, brought to 1.0 M (NH_4_)_2_SO_4_, and subjected to Source 15ISO (GE Healthcare) hydrophobic interaction chromatography with a 1.0 to 0.2 M (NH_4_)_2_SO_4_ gradient in 20 mM sodium acetate buffer at pH 5.5 (buffer C). The Xyl10A fractions that showed synergistic activity toward Avicel with both F2 and F3 were pooled, desalted, and concentrated.

The F2 sample, which included Cel6A, was applied to a Source 15S cation-exchange column equilibrated with buffer B. The flow-through was pooled, brought to 0.8 M (NH_4_)_2_SO_4_, and subjected to Source 15ISO column chromatography with a 0.8 to 0.24 M (NH_4_)_2_SO_4_ gradient in buffer C. The Cel6A fractions having synergistic activity with F3 were pooled, desalted, and concentrated.

The F3 sample, which included Cel7A and Bgl3A, was applied to a Source 15S cation-exchange column equilibrated with buffer B and then eluted with the same buffer. The flow-through fractions were pooled, brought to 1.2 M (NH_4_)_2_SO_4_, and subjected to Source 15ISO column chromatography with a 1.2 to 0 M (NH_4_)_2_SO_4_ gradient in buffer C. Cel7A peak fractions and Bgl3A peak fractions having synergistic activity with F2 were separately pooled and desalted. The Cel7A fractions were further purified by affinity chromatography on a 4-aminophenyl-β-d-cellobioside-conjugated Affi-Gel 10 column (Bio-Rad, Hercules, California, United States) followed by Source 15Q anion-exchange column chromatography to remove β-glucosidase contamination [25]. The Bgl3A fractions were applied to a Source 15Q column equilibrated with buffer A and eluted with a linear gradient of 0 to 0.1 M NaCl. Major single peak fractions were pooled, desalted, and concentrated.

The F4 sample, which included Cel5A and Cel7B, was applied to a Source 15S cation-exchange column equilibrated with buffer B and then eluted with the same buffer. The flow-through fractions were pooled, brought to 1.0 M (NH_4_)_2_SO_4_, and subjected to Source 15ISO column chromatography with a 1.0 to 0.25 M (NH_4_)_2_SO_4_ gradient in buffer C. The peaks having synergistic activity with both F2 and F3 were pooled. The desalted sample was applied to a Source 15Q column equilibrated with buffer A and eluted with a linear gradient of 0.1 to 0.15 M NaCl. The two active fractions corresponding to Cel5A and Cel7B were separately pooled, desalted, and concentrated. The Cel5A fractions were then brought to 0.8 M (NH_4_)_2_SO_4_ and applied to a Source 15Phe hydrophobic interaction column (GE Healthcare) with a 0.8 to 0 M (NH_4_)_2_SO_4_ gradient in buffer C. The Cel5A fractions were pooled, desalted, and concentrated.

All purified enzymes gave a single band in SDS-PAGE and were preserved in a 20 mM sodium acetate buffer (pH 5.5) containing 0.01% NaN_3_ at 4°C. The protein concentration was determined with a bicinchoninic acid protein assay kit (Thermo Scientific, Rockford, Illinois, United States), using bovine serum albumin (Thermo Scientific) as the protein standard. The molecular weights of native enzymes were determined using a TSKgel G3000SW_XL_ size-exclusion column (7.8 mm internal diameter × 30 cm) (Tosoh, Tokyo, Japan) on a high-performance liquid chromatography system equipped with a UV detector (RI-2070Plus, JASCO, Tokyo, Japan). The mobile phase was a 20 mM sodium phosphate buffer (pH 6.3) containing 0.3 M NaCl, and the flow rate was 0.5 mL/min at a column temperature of 28°C. The eluted proteins were detected at 280 nm. A calibration curve was plotted using the standard proteins in a gel filtration calibration kit (GE Healthcare).

### Assay for enzyme purification

The assay for synergistic hydrolysis of Avicel was carried out in 1 mL of 50 mM sodium acetate buffer (pH 5.0). Enzyme sample (25 μg) from chromatogram peak fractions was added to a mixture containing 20 mg of Avicel PH-101 (11365 Fluka, Sigma-Aldrich, St. Louis, Missouri, United States) and 25 μg of the F2 or F3 sample prepared by Source 15Q column chromatography of the culture filtrate (Figure 1). The reaction mixture was incubated in a 1.5-mL tube at 45°C for two hours on a rotator. The concentration of reducing sugars was determined using 3,5-dinitrosalicylic acid and compared with that in the control mixture containing 20 mg of Avicel PH-101 and 50 μg of the F2 or F3 sample. Synergism (%) was calculated as follows:

$$ \left(\mathrm{Reducing}\kern0.5em \mathrm{sugars}\kern0.5em \mathrm{in}\kern0.5em \mathrm{the}\kern0.5em \mathrm{reaction}\kern0.5em \mathrm{mixture}/\mathrm{reducing}\kern0.5em \mathrm{sugars}\kern0.5em \mathrm{in}\kern0.5em \mathrm{the}\kern0.5em \mathrm{control}\kern0.5em \mathrm{mixture}\right)\times 100 $$

The samples showing more than 100% synergism were pooled and used for further purification steps.

### Enzyme activity assays

All enzyme assays were carried out in 1 mL of 50 mM sodium acetate buffer (pH 5.0) at 45°C. Avicelase activity was determined by estimating the concentration of reducing sugars released after 60 minutes of reaction with 20 mg of Avicel PH-101. Carboxymethyl cellulase and xylanase activities were measured by assaying the reducing sugars released after 30 minutes of reaction with 1% (w/v) CMC (low viscosity) and 1% (w/v) birch-wood xylan, respectively. One unit of enzyme activity was defined as the amount of enzyme that catalyzed the formation of 1 μmol of reducing sugar per minute. The enzyme activities against 1 mM p-nitrophenol-based chromogenic glycosides (p-nitrophenyl-β-d-lactoside, p-nitrophenyl-β-d-glucoside, and p-nitrophenyl-β-d-xyloside) were assayed as described elsewhere [46]. One unit of enzyme activity was defined as the amount of enzyme that catalyzed the formation of 1 μmol of p-nitrophenol per minute.

### Hydrolysis of pretreated corn stover

The PCS slurry was washed with distilled water to remove acids and soluble compounds. The washed solid fraction was suspended in 50 mM acetate buffer (pH 5.0), and a final concentration of 3% (w/v) was used for enzymatic hydrolysis. In the standard assay using purified enzymes, PCS hydrolysis was carried out at a protein loading of 2.55 mg/g glucan, consisting of 40 μg of the mixed cellulolytic enzymes and 5 μg of Bgl3A in a final volume of 1 mL. The reaction mixture (pH 5.0) was incubated at 45°C for 48 hours on a rotator. Glucose released in the supernatant of the mixture was determined using a BF-5 biosensor with a glucose oxidase electrode (Oji Scientific Instruments, Amagasaki, Japan). The CF-2612 culture supernatant and a commercial cellulase (Cellic CTec2, Novozymes NA., Franklinton, North Carolina, United States) were used for PCS hydrolysis for comparison with the optimized cellulolytic enzyme mixture. All PCS hydrolysis experiments were run in duplicate and assayed twice (n =4).

Design-Expert software (Stat-Ease Inc., Minneapolis, Minnesota, United States) was used for experimental design and analysis to optimize the composition of the four cellulolytic enzymes (Cel5A, Cel6A, Cel7A, and Xyl10A) for PCS hydrolysis. An augmented quadratic design was used throughout, as described by Banerjee *et al.* [47]; the simplex-lattice design containing four components required 15 reactions. The minimum enzyme proportions were set to 20% for Cel6A and Cel7A and 5% for Cel5A and Xyl10A. All data were analyzed by ANOVA to develop a statistically-based predictive model and the *F* ratio, *P* value, *R*^2^, adjusted *R*^2^, predicted *R*^2^, and adequate precision were calculated [8].

### Identification of cellulolytic enzymes

Identification of all purified enzymes was conducted by Stanford Mass Spectrometry Services (Stanford University, Stanford, California, United States). Trypsin-digested peptide fragments of the purified enzymes were analyzed using an electrospray ionization quadruple-time-of-flight mass spectrometer (Micromass UK, Manchester, United Kingdom). Scaffold 3 (Proteome Software, Portland, Oregon, United States) was used to validate protein identifications by matching the mass spectra with *Aspergillus*, *Talaromyces*, and *Hypocrea* peptide sequences in the NCBI database.

Trypsin-digested peptide fragments of purified Cel5A and Cel7B were further analyzed by matrix-assisted laser desorption ionization time-of-flight mass spectrometry (Voyager-DE STR, Applied Biosystems, Foster City, California, United States). The fragment masses were analyzed using the MASCOT server (Matrix Science, London, United Kingdom) and compared with amino acid sequence translations of all open reading frames in our in-house *T. cellulolyticus* draft genome.

### Protein thermal shift assay

The fluorescence-based thermal shift assay was performed using a real-time PCR detection system (CFX Connect, Bio-Rad) [48]. SYPRO orange dye (Invitrogen, Carlsbad, California, United States) was added to protein samples and the samples were heated at 0.5°C per five seconds from 25 to 95°C. The fluorescence intensity (excitation/emission: 450 to 490 nm/560 to 580 nm) was measured every 0.5°C. Thermal midpoint (*T*_m_) values of proteins were determined with the CFX Manager Program (Bio-Rad) based on calculation of the negative first derivative.

## Abbreviations

AFEX: ammonia fiber expansion
ANOVA: analysis of variance
Bgl3A: glycosyl hydrolase family 3 β-glucosidase
CBH: cellobiohydrolase
Cel5A: glycosyl hydrolase family 5 endoglucanase
Cel6A: glycosyl hydrolase family 6 cellobiohydrolase II
Cel7A: glycosyl hydrolase family 7 cellobiohydrolase I
Cel7B: glycosyl hydrolase family 7 endoglucanase
CMC: carboxymethyl cellulose
EG: endoglucanase
GH: glycosyl hydrolase
MALDI-TOF MS: matrix-assisted laser desorption ionization time-of-flight mass spectrometry
MES: 2-(N-morpholino)ethanesulfonic acid
MS/MS: tandem mass spectrometry
PCS: pretreated corn stover
PNP-glucose: p-nitrophenyl-β-d-glucoside
PNP-lactose: p-nitrophenyl-β-d-lactoside
PNP-xylose: p-nitrophenyl-β-d-xyloside
SD: standard deviation
*T*_m_: thermal midpoint
Xyl10A: glycosyl hydrolase family 10 xylanase
